# Identification of non-coding RNAs embracing *microRNA-143/145 *cluster

**DOI:** 10.1186/1476-4598-9-136

**Published:** 2010-06-02

**Authors:** Akio Iio, Yoshihito Nakagawa, Ichiro Hirata, Tomoki Naoe, Yukihiro Akao

**Affiliations:** 1 Department of Medical Oncology, Gifu International Institute of Biotechnology, 1-1 Naka-Fudogaoka, Kakamigahara, Gifu 504-0838, Japan; 2 Department of Gastroenterology, Fujita Health University, School of Medicine, 1-98 Dengakugakubo, Kutsukake-cho, Toyoake, Aichi 470-1192, Japan; 3 Department of Hematology and Oncology, Nagoya University, Graduate School of Medicine, 65 Tsurumai-cho, Showa-ku, Nagoya, Aichi 466-8550, Japan; 4 United Graduate School of Drug Discovery and Medical Information Sciences, Gifu University, Yanagido, Gifu, Gifu 501-1193, Japan

## Abstract

In a variety of cancers, altered patterns of microRNA (miRNA) expression are reported and may affect the cell cycle and cell survival. Recent studies suggest that the expression level of miRNAs that act as tumor suppressors is frequently reduced in cancers because of chromosome deletions, epigenetical changes, aberrant transcription and disturbances in miRNA processing. *miR-143 *and -*145*, which are located approximately 1.3 kb from each other at chromosome 5q33, are highly expressed in several tissues, but down-regulated in most cancers. However, the mechanism of this down-regulation has not been investigated in detail. Here, we show that both miRNAs were expressed well under the same control program in human tissues, but were down-regulated equally in the most of the cancer cell lines tested. Then we identified the host gene encoding both miRNAs. The transcripts of this gene were approximately 11, 7.5, and 5.5 kb long; and the expression of these transcripts was coordinated with that of its resident miRNAs and down-regulated in the cancer cell lines tested as well as in colorectal cancer tissue samples. These data demonstrate that the host gene can function as a primary miRNA transcript and suggest that the down-regulation of host gene expression caused the low-expression of its encoded *microRNAs-143 *and -*145 *in human cancer cell lines and in cancer tissues.

## Findings

MicroRNAs (miRNAs) are tiny, endogenously expressed noncoding RNAs (18-25 nucleotides in length) that act as crucial posttranscriptional regulators of gene expression [1-3]. For several miRNAs, their participation in essential biological processes has been proved, such as in cell proliferation control, cell lineage fate decision, cell survival, tissue patterning for development, and cell metabolism [4]. Cancer is a very complex genetic disease characterized by alterations in genes encoding oncogenic and tumor-suppressor proteins [5]. Recently, it has been noted that the expression profiles of miRNAs can be used for classification, diagnosis, and prognosis of human malignancies; and the deletion or amplification of the locus encoding an miRNA in a variety of cancers has been reported. Altered patterns of miRNA expression may affect cell-cycle and survival programs and be involved in tumor initiation and progression. We previously found that *microRNA-143 *(*miR-143*) and -*145 *(*miR-145*) were down-regulated in colon cancers [6,7], gastric cancers [8], chronic lymphocytic leukemias, and B cell lymphomas [9], and in several human cancer cell lines [7]. Several groups also reported the down-regulation of both of these miRNAs in many other types of cancers, such as bladder cancers and their cell lines [10,11], cervical cancers and their cell lines [12], colorectal cancers [13-16], nasopharyngeal carcinoma [17], and prostate cancer [18]. Furthermore, such abnormal expression was found not only in malignant cells but also in cells in premalignant stages such as colon adenoma cells [13,19]. The introduction of the mature type of either *miR-143 *or -*145 *into colon cancer cells [6,7,20], B cell lymphoma [9], and gastric cancer cells [8,21] results in a significant growth inhibition that occurs in a dose-dependent manner; and the target genes, *ERK5 *[22] and *KRAS *[20] for *miR-143 *and *IRS-1 *[23] and *c-myc *[21] for *miR-145*, were posttranscriptionally down-regulated. Taken together, these findings suggest that *miR-143 *and -*145 *act as tumor suppressors and provide an important clue in the study of the mechanism of tumor initiation and progression involving miRNAs.

In the present study, we identified non-coding RNAs carrying an *miR-143 *and -*145 *cluster (*NCR143/145*: Non-coding RNA encoding *miR-143/145*) and investigated the expression of *NCR143/145 *in all cancer cell lines tested. Importantly, the down-regulation of this host gene expression caused the low expression of both miRNAs in human cancer cell lines, which could lead to tumor development and progression.

## Expression of *miR-143 and -145*

**Figure 1:**
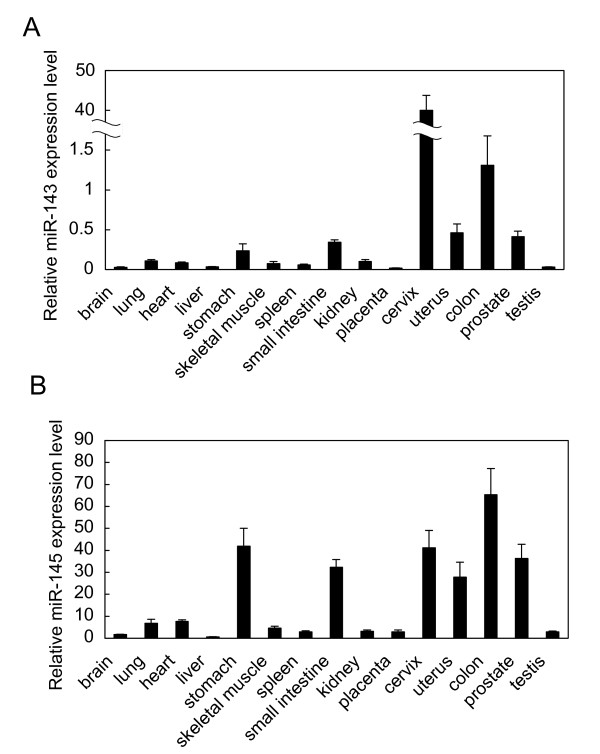
**Real-time RT-PCR analysis of mature *miR-143 *and -*145 *expression in human normal tissues**. Total RNAs from human normal tissues were purchased from Biochain (Hayward, USA) and Takara (Otsu, Japan). Expression of both miRNAs in each sample was detected by using a TaqMan microRNA reverse transcription kit and TaqMan microRNA Assay Kit (Applied Biosystems, Foster City, USA) and normalized with *RNU6B*. Calculation of the Ct value was done by using the second derivative maximum method, and relative quantification was analyzed by the comparative Ct (ΔCt) method. All reactions were run in triplicate. Relative *miR-143 ***(A) **and -*145***(B) **expression levels (value of 2^-ΔCt(miR-RNU6B)^) are indicated on the left axis, with error bars indicating the standard deviation for these analyses.

We examined the expression levels of mature *miR-143 *and -*145 *in human normal tissues by performing TaqMan microRNA assays (Fig. 1). In human normal tissues, *miR-143 *and -*145 *showed good expression in stomach, intestine, cervix, uterus, colon, and prostate (Fig. 1A, B). Whereas, in cancer cell lines, they were expressed at an extremely low level compared with that in human normal cell lines (Additional file 1 - Figure S1). Compared with their expression in corresponding normal tissues, the expression levels of both miRNAs were obviously down-regulated in all cancer cell lines and cancer tissue samples tested, just as many groups had previously reported [6-18]. Such a similar expression pattern of them indicates that the expression of both miRNAs may be regulated by a similar mechanism. Additionally, the DNA loci of both miRNAs are very close to each other, within 1.3 kb, which led us to speculate that both precursors may originate from the same primary transcript. Genomic PCR spanning this region demonstrates the fragment in most of the cancer cell lines tested [6,7,9]. Therefore, we decided to isolate the gene that carried both miRNAs in a cluster.

## Identification of non-coding RNA carrying the *miR-143/145 *cluster

**Figure 2:**
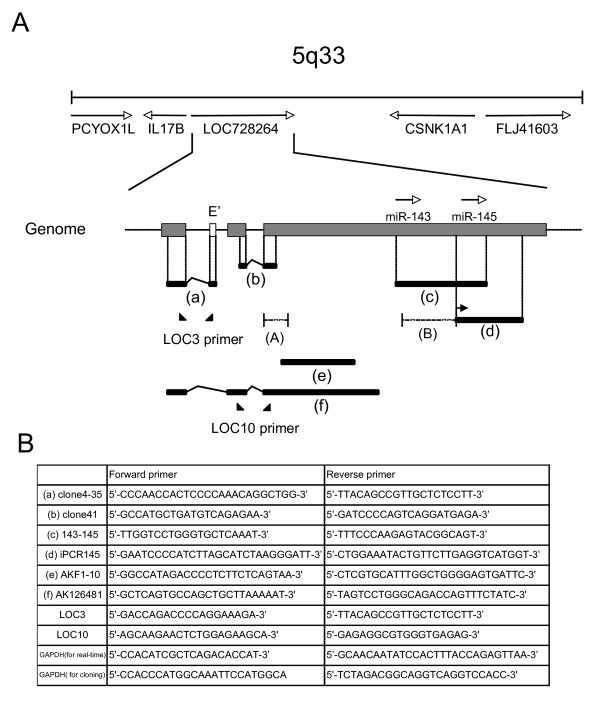
**Identification and characterization of the host gene encoding *miR-143 *and -*145***. (**A**) Scheme of the cytogenetic map of chromosomal region 5q33. RT-PCR cloning was performed by using the primer sets (**B**) that covered the predicted first exon-containing region (clone4-35;a) of LOC728264 [GeneBank:NR027180], predicted intron-spanning region (clone41;b), both *miR-143 *and -*145 *regions (143-145;c), predicted pre-miR-145 region (iPCR145;d), AKF1-10 region (e), and AK126481 region (GeneBank;f) from human placenta and uterus cDNA, and cDNA library. The positions of neighboring genes, *PCYOX1L*, *IL17B*, *CSNK1A1*, and *FLJ41603 *are also shown as references. The real-time RT-PCR primer sets (LOC3 and 10) specific for this gene are indicated by the arrowheads. The open box (E') represents a novel exon derived from a cDNA clone (a). This exon is not shown in the NCBI database. Region "A" is a cardiac-specific enhancer reported by Cordes [29], and region "B" is *p53 *and/or *Oct4*-dependent *miR-145 *specific promoter reported by Sachdeva [21] and Xu [24].

First, we carried out RT-PCR and inverted PCR cloning method using human placenta and uterus cDNA, and a placental cDNA library, and isolated each of the cDNA clones designated in Figure 2. The 143-145 clone was 2.2 kb long and detected in human tissues such as uterus, prostate, and testis by RT-PCR (data not shown). The iPCR145 clone, which encoded *miR-145*, was 1.7 kb long and corresponded to the transcriptional unit for only *miR-145 *identified by Sachdeva [21] and Xu [24]. Clone 41 was 373 bp long (Fig. 2B) and amplified at a high level in human normal tissues by semi-qRT-PCR, but hardly amplified in several cancer cell lines examined (data not shown). Clone AK126481 was 3.8 kb long and identical to AK126481 in GeneBank, and clone AKF1-10 was 1.8 kb long and overlapped with AK126481. Clone 4-35 was 129 bp long and contained a part of the predicted first exon and novel second exon (Fig. 2A). At the upstream of this predicted first exon, hypothetical transcriptional start site was localized, which was shown by Fujita [25]. Also, the homolog of this gene (*IE 1071*) and promoter region were cloned in the mouse by Ebisuya [26] and shown to be comparatively conserved between human and mouse. This indicates that the predicted transcriptional start site near the sequence of clone 4-35 is a putative promoter region of the *miR-143 *and -*145*-encoding gene.

**Figure 3:**
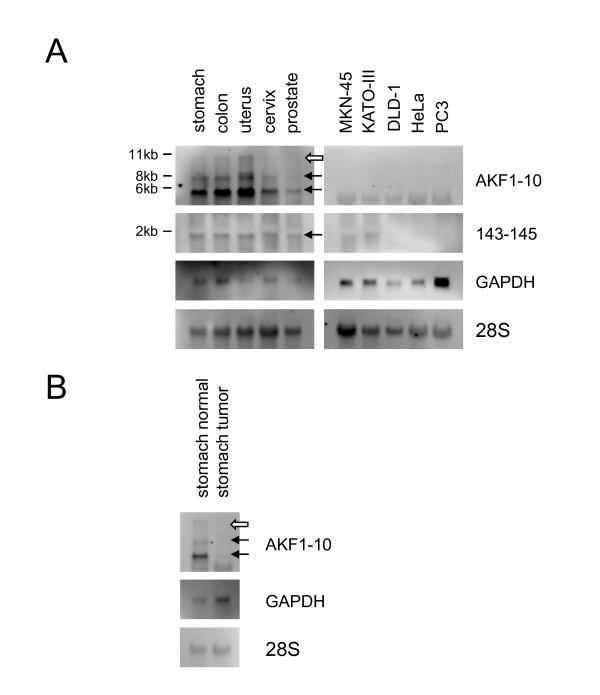
**Northern blot analysis of the host gene encoding *miR-143 *and -*145***. Total RNAs (5 μg for human normal tissues, cancer tissue, and cancer cell lines [human stomach tumor RNA was purchased from Takara]) were separated on a 1%(w/v) formaldehyde agarose gel and then blotted onto Hybond N+ nylon membranes (Amersham Biosciences, Piscataway, USA). RNA probes were synthesized from template cDNAs by using a MAXIscript kit (Ambion, Austin, USA) incorporating Digoxigenin-11-UTP (Roche, Penzberg, Germany). Northern blots were hybridized with the AKF1-10 clone (**A**, upper panel; refer to Fig. 2), 143-145 clone (**A**, 2nd panel), *GAPDH *(**A**, 3rd panel; **B**, middle panel), and 28 S rRNA (**A **and **B**, under panel). The primary transcript is marked by the open arrow and other transcripts are marked by closed arrows.

Next, we performed Northern blot analysis to look for the transcripts that originated from the host gene encoding *miR-143 *and -*145*. The large transcript (11 kb: open arrow) and 3 or 4 transcripts (7.5, 5.5, and 1.9 kb: closed arrows) were detected (Fig. 3). The 11-kb transcript was hybridized with 6 probes (Fig. 2, a-f; Fig. 3), and the 1.9-kb one was only detected by 143-145 (Fig. 3) and iPCR145 probes (data not shown), and not detected by the 4-35, 41, AKF1-10 or AK126481 probes (data not shown). These results indicate that the host gene was firstly transcribed into the 11-kb transcript and then processed to the mature *miR-143 *and -*145 *via 7.5 and 5.5 kb processed variant transcripts. This gene is the non-coding RNA shown by Ebisuya to be subject to splicing [26]. Also, in human normal tissues, *miR-145 *was consistently expressed at higher levels than *miR-143 *(Fig. 1A, B). Apart from both miRNAs being produced from the 11-kb transcript, *miR-145 *would also be generated from the 1.9-kb transcript. It is thought that this expression of *miR-145 *is regulated by a different mechanism dependent on *p53 *[21] and/or *Oct4 *[24]. In our preliminary experiments, the expression of host gene and its promoter activity were p53-independent in p53-mutated cancer cell lines (MKN-45 and DLD-1, data not shown). This finding of p53 independency raises the possibility that p53-dependent gene expression and other pathways are abrogated in p53-mutated cancer cell lines. We are currently investigated this point in our laboratory.

## Regulation of *NCR143/145 *expression in cancers

**Figure 4:**
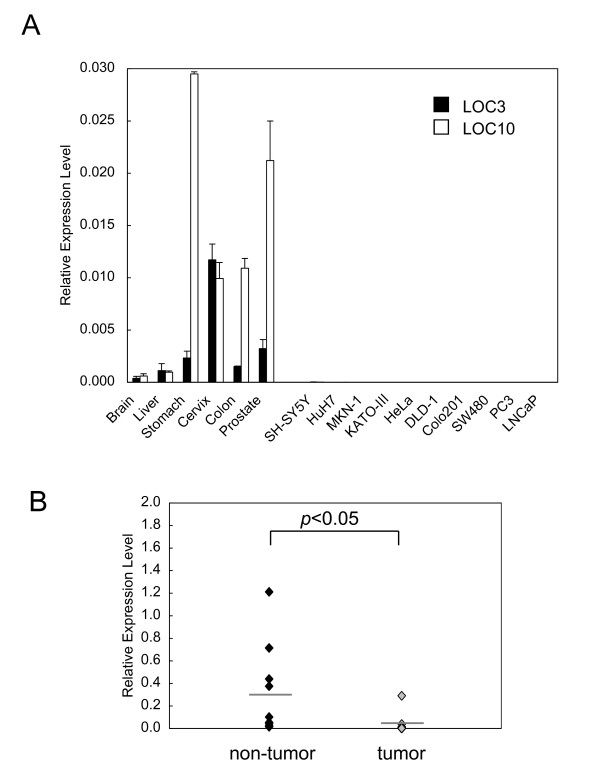
**Real-time RT-PCR analysis of the host gene encoding *miR-143 *and -*145***. Real-time RT-PCR analysis was performed on human normal tissues and cancer cell lines (**A**) and human colon adenocarcinoma samples (**B**) by using host gene-specific primers. Ten pairs of colon samples, including 10 human colon adenocarcinoma tissue samples and 10 matched normal colon tissue samples, were obtained from Osaka Medical College Hospital (Takatsuki, Osaka, Japan) and Fujita Health University Hospital (Toyoake, Aichi, Japan) with patients' informed consent. Collection and distribution of the samples were approved by each of the appropriate institutional review boards. The category of colon samples was confirmed by pathological analysis. Total RNA was extracted from the colon samples by using TRIzol (Invitrogen, Carlsbad, USA) according to the manufacturer's protocol. Expression levels of the host gene in each sample (LOC3 and LOC10, refer to Fig. 2) were detected by using a Superscript III reverse transcription kit (Invitrogen, Carlsbad, USA) and SYBR Premix Ex Taq (Takara, Otsu, Japan) and normalized with *GAPDH*. Ruled lines present the medians of samples. Data were analyzed by using Student's *t*-test. A value of *p *< 0.05 was considered significant.

Most miRNAs located within protein-coding or non-coding genes are transcriptionally linked to the expression of their host genes [27]. In order to investigate the coordinated expression of the host gene identified in this study with mature *miR-143 *and -*145*, we performed real-time RT-PCR analysis by using the host gene-specific primer set shown in Fig. 2. In human normal tissues, the host gene was highly expressed, as were both miRNAs; but in the corresponding cancer cell lines, the signal was hardly detected (Fig. 4A), though the host gene and both miRNAs were highly expressed in normal human cell lines (Additional files 1 - Figure S1 & S2). Also in human cancer tissues, the host gene was down-regulated compared with its expression in normal human tissues (Figs. 3, 4B and additional file 1 - Figure S3). As a result, the down-regulation of host gene *NCR143/145 *expression caused low expression of both mature miRNAs in human cancer cell lines. Thus, the aberrant transcription of *NCR143/145 *could contribute to the low expression of *miR-143 *and -*145*.

The expression level of miRNAs that act as tumor suppressors is frequently reduced in cancers because of chromosome deletions, epigenetical changes, aberrant transcription, and disturbances in miRNA processing. Michael et al. [13] reported that in colorectal cancers the decreased levels of mature *miR-143 *and -*145 *were due to reduced *Dicer*-processing activities. In our study, the activity and expression of *Dicer *and *RISC *proteins seemed to be intact in colorectal cancer cells, because the expression levels of *miR-143 *and -*145 *were up-regulated by stimulation with a growth inhibitor [7]. Therefore, we propose that the inadequate expression of *miR-143 *and -*145 *was due to the perturbation of transcription and/or that of the another processing enzyme, *Drosha*, which causes the transit from primary miRNAs to precursor ones. Recently, it was reported that *p53 *interacts with the *Drosha *processing complex through association with *DDX5 *and facilitates the processing of primary miRNAs to precursor ones [28]. That report also indicated that mutant *p53 *interfered with these processing activities. These findings suggest that the inappropriate *p53*-dependent modulation of miRNA biogenesis also affects the expression of mature miRNAs in cancer cells. But, in our study, *Drosha *expression and its processing activity seemed to be normal in *p53*-mutated MKN-45 and K562 cells (data not shown).

To exclude the possibility that the DNA of the loci and histone were epigenetically methylated, in earlier studies [6,7,9] we examined by qRT-PCR the expression of both miRNAs in DLD-1, SW480 and EBV-transformed L25 cells after treatment of the cells with 5-aza-2'-deoxycytidine and tricostatin A. As a result, the levels of both miRNAs were almost unchanged by either treatment [6,7,9].

To confirm the presence of the genomic locus including both miRNAs at chromosome 5q33, we carried out genomic PCR on several cancer cell lines (Additional file 1 - figure S4) [6,7,9]. In HeLa, U937, and PC3 cells, one allele of the locus might have been deleted. This locus is frequently involved in chromosome copy number loss in various types of cancers including non-small cell lung cancer and gastric cancer according to the CGH database http://www.cghtmd.jp/CGHDatabase/index_j.jsp. Therefore, further detailed cytogenetic study will be needed to understand the mechanism of *miR-143 *and -*145 *down-regulation in many cancer cell lines.

## Abbreviations

ERK5: extracellular signal-regulated kinase 5; KRAS: v-Ki-ras2 Kirsten rat sarcoma viral oncogene homolog; IRS-1: insulin receptor substrate 1; Oct4: Octamer-4; RISC: RNA-induced silencing complex; DDX5: DEAD box polypeptide 5; EBV: Epstein-Barr virus; CGH: Comparative Genomic Hybridization; RNU6B: U6 small nuclear 2 RNA; Ct: cycle threshold: PCYOX1L: prenylcysteine oxidase 1 like; IL17B: interleukin 17B; CSNK1A1: casein kinase 1 alpha 1; UTP: uridine triphosphate; GAPDH: glyceraldehyde-3-phosphate dehydrogenase; rRNA: ribosomal RNA;

## Competing interests

The authors declare that they have no competing interests.

## Authors' contributions

AI and YA conceived and planned the experiments. TN and YA provided the human cancer cell lines. YN and IH collected the clinical specimens. AI performed all experiments. All authors read and approved the manuscript.

## Supplementary Material

Additional file 1 **Supplementary figures. Figure S1: Real-time RT-PCR analysis of mature *miR-143 *and -*145 *expression in human cell lines**. Relative *miR-143 *(A) and -*145 *(B) expression levels are indicated on the left axis by using the comparative ΔCt method (value of 2^-ΔCt(miR-RNU6B)^). **Figure S2: Real-time RT-PCR analysis of *NCR143/145 *expression in human cancer cell lines and normal cell lines**. The relative expression level of *NCR143/145 *in human cancer cell lines was compared with that in human normal cell lines (WI-38 and IMR-90) by using the LOC10 primer set (see Fig. 2). **Figure S3: Real-time RT-PCR analysis of *NCR143/145 *expression in human stomach**. The relative expression level of *NCR143/145 *in human stomach tumor was compared with that in normal human stomach by using the LOC10 primer set (see Fig. 2). **Figure S4: Confirmation of the presence of genomic loci of *miR-143 *and -*145 *at chromosome 5q33 by genomic PCR**. We extracted genomic DNAs from 2 cell lines and normal human oral squamous cells by using DNAzol (Invitrogen, Carlsbad, USA) and used them for PCR. The 143-145 primer set was used for genomic loci of miRs-143 and -145 (see Fig. 2B). The genomic locus of GAPDH was used as an internal control. Click here for file

## References

[B1] Bartel DP MicroRNAs: genomics, biogenesis, mechanism, and function Cell 2004 116 281 97 10.1016/S0092-8674(04)00045-5 14744438

[B2] Pillai RS MicroRNA function: multiple mechanisms for a tiny RNA? RNA 2005 11 1753 61 10.1261/rna.2248605 16314451 PMC1370863

[B3] Nilsen TW Mechanisms of microRNA-mediated gene regulation in animal cells Trends Genet 2007 23 243 9 10.1016/j.tig.2007.02.011 17368621

[B4] Harfe BD MicroRNAs in vertebrate development Curr Opin Genet Dev 2005 15 410 5 10.1016/j.gde.2005.06.012 15979303

[B5] Calin GA Croce CM MicroRNA-cancer connection: the beginning of a new tale Cancer Res 2006 66 7390 4 10.1158/0008-5472.CAN-06-0800 16885332

[B6] Akao Y Nakagawa Y Naoe T MicroRNAs 143 and 145 are possible common onco-microRNAs in human cancers Oncol Rep 2006 16 845 50 16969504

[B7] Akao Y Nakagawa Y Naoe T MicroRNA-143 and -145 in colon cancer DNA Cell Biol 17504027 10.1089/dna.2006.0550

[B8] Takagi T Iio A Nakagawa Y Naoe T Tanigawa N Akao Y Decreased expression of microRNAs-143 and -145 in human gastric cancers Oncology 2009 77 12 21 10.1159/000218166 19439999

[B9] Akao Y Nakagawa Y Kitade Y Kinoshita T Naoe T Down-regulation of micoRNAs-143 and -145 in B-cell malignancies Cancer Sci 2007 98 1914 20 10.1111/j.1349-7006.2007.00618.x 17892514 PMC11158757

[B10] Lin T Dong W Huang J Pan Q Fan X Zhang C Huang L MicroRNA-143 as a tumor suppressor for bladder cancer J Urol 2009 181 1372 80 10.1016/j.juro.2008.10.149 19157460

[B11] Dyrskjøt L Ostenfeld MS Bramsen JB Silahtaroglu AN Lamy P Ramanathan R Fristrup N Jensen JL Andersen CL Zieger K Kauppinen S Ulhøi BP Kjems J Borre M Ørntoft TF Genomic profiling of microRNAs in bladder cancer: miR-129 is associated with poor outcome and promotes cell death in vitro Cancer Res 2009 69 4851 60 10.1158/0008-5472.CAN-08-4043 19487295

[B12] Wang X Tang S Le SY Lu R Rader JS Meyers C Zheng Z-M Aberrant expression of oncogenic and tumor-suppressive microRNAs in cervical cancer is required for cancer cell growth PLoS One 2008 3 e2557 10.1371/journal.pone.0002557 18596939 PMC2438475

[B13] Michael MZ O' Connor SM van Holst Pellekaan NG Young GP James RJ Reduced accumulation of specific microRNAs in colorectal neoplasia Mol Cancer Res 2003 1 882 91 14573789

[B14] Slaby O Svoboda M Fabian P Smerdova T Knoflickova D Bednarikova M Nenutil R Vyzula R Altered expression of miR-21, miR-31, miR-143 and miR-145 is related to clinicopathologic features of colorectal cancer Oncology 2007 72 397 402 10.1159/000113489 18196926

[B15] Wang CJ Zhou ZG Wang L Yang L Zhou B Gu J Chen HY Sun XF Clinicopathological significance of microRNA-31, -143 and -145 expression in colorectal cancer Dis Markers 2009 26 27 34 19242066 10.3233/DMA-2009-0601 PMC3833327

[B16] Motoyama K Inoue H Takatsuno Y Tanaka F Mimori K Uetake H Sugihara K Mori M Over- and under-expressed microRNAs in human colorectal cancer Int J Oncol 2009 34 1069 75 19287964 10.3892/ijo_00000233

[B17] Chen HC Chen GH Chen YH Liao WL Liu CY Chang KP Chang YS Chen SJ MicroRNA deregulation and pathway alterations in nasopharyngeal carcinoma Br J Cancer 2009 100 1002 11 10.1038/sj.bjc.6604948 19293812 PMC2661776

[B18] Tong AW Fulgham P Jay C Chen P Khalil I Liu S Senzer N Eklund AC Han J Nemunaitis J MicroRNA profile analysis of human prostate cancers Cancer Gene Ther 2009 16 206 16 18949015 10.1038/cgt.2008.77

[B19] Akao Y Nakagawa Y Hirata I Iio A Ito T Kojima K Nakashima R Kitade Y Naoe T Role of anti-oncomirs miR-143 and -145 in human colorectal tumors Cancer Gene Ther 2010 17 398 408 10.1038/cgt.2009.88 20094072

[B20] Chen X Guo X Zhang H Xiang Y Chen J Yin Y Cai X Wang K Wang G Ba Y Zhu L Wang J Yang R Zhang Y Ren Z Zen K Zhang J Zhang CY Role of miR-143 targeting KRAS in colorectal tumorigenesis Oncogene 2009 28 1385 1392 10.1038/onc.2008.474 19137007

[B21] Sachdeva M Zhu S Wu F Wu H Walia V Kumar S Elble R Watabe K Mo YY p53 represses c-Myc through induction of the tumor suppressor miR-145 Proc Natl Acad Sci USA 2009 106 3207 12 10.1073/pnas.0808042106 19202062 PMC2651330

[B22] Akao Y Nakagawa Y Iio A Naoe T Role of microRNA-143 in Fas-mediated apoptosis in human T-cell leukemia Jurkat cells Leukemia Res 2009 33 1530 1538 10.1016/j.leukres.2009.04.019 19464056

[B23] Shi B Sepp-Lorenzino L Prisco M Linsley P deAngelis T Baserga R Micro RNA 145 targets the insulin receptor substrate-1 and inhibits the growth of colon cancer cells J Biol Chem 2007 282 32582 32590 10.1074/jbc.M702806200 17827156

[B24] Xu N Papagiannakopoulos T Pan G Thomson JA Kosik KS MicroRNA-145 regulates OCT4, SOX2, and KLF4 and represses pluripotency in human embryonic stem cells Cell 2009 137 647 58 10.1016/j.cell.2009.02.038 19409607

[B25] Fujita S Iba H Putative promoter regions of miRNA genes involved in evolutionarily conserved regulatory systems among vertebrates Bioinformatics 2008 24 303 8 10.1093/bioinformatics/btm589 18055479

[B26] Ebisuya M Yamamoto T Nakajima M Nishida E Ripples from neighbouring transcription Nat Cell Biol 2008 10 1106 13 10.1038/ncb1771 19160492

[B27] Rodriguez A Griffiths-Jones S Ashurst JL Bradley A Identification of mammalian microRNA host genes and transcription units Genome Res 2004 14 1902 10 10.1101/gr.2722704 15364901 PMC524413

[B28] Suzuki HI Yamagata K Sugimoto K Iwamoto T Kato S Miyazono K Modulation of microRNA processing by p53 Nature 2009 460 529 33 10.1038/nature08199 19626115

[B29] Cordes KR Sheehy NT White MP Berry EC Morton SU Muth AN Lee TH Miano JM Ivey KN Srivastava D miR-145 and miR-143 regulate smooth muscle cell fate and plasticity Nature 2009 460 705 10 19578358 10.1038/nature08195 PMC2769203

