# Supplementary figures and images for: Identification of non-coding RNAs embracing microRNA-143/145 cluster
Source: Mol Cancer. 2010 Jun 2;9:136. doi: 10.1186/1476-4598-9-136 (PMC2903500; doi:10.1186/1476-4598-9-136)

# Figure S1

## A

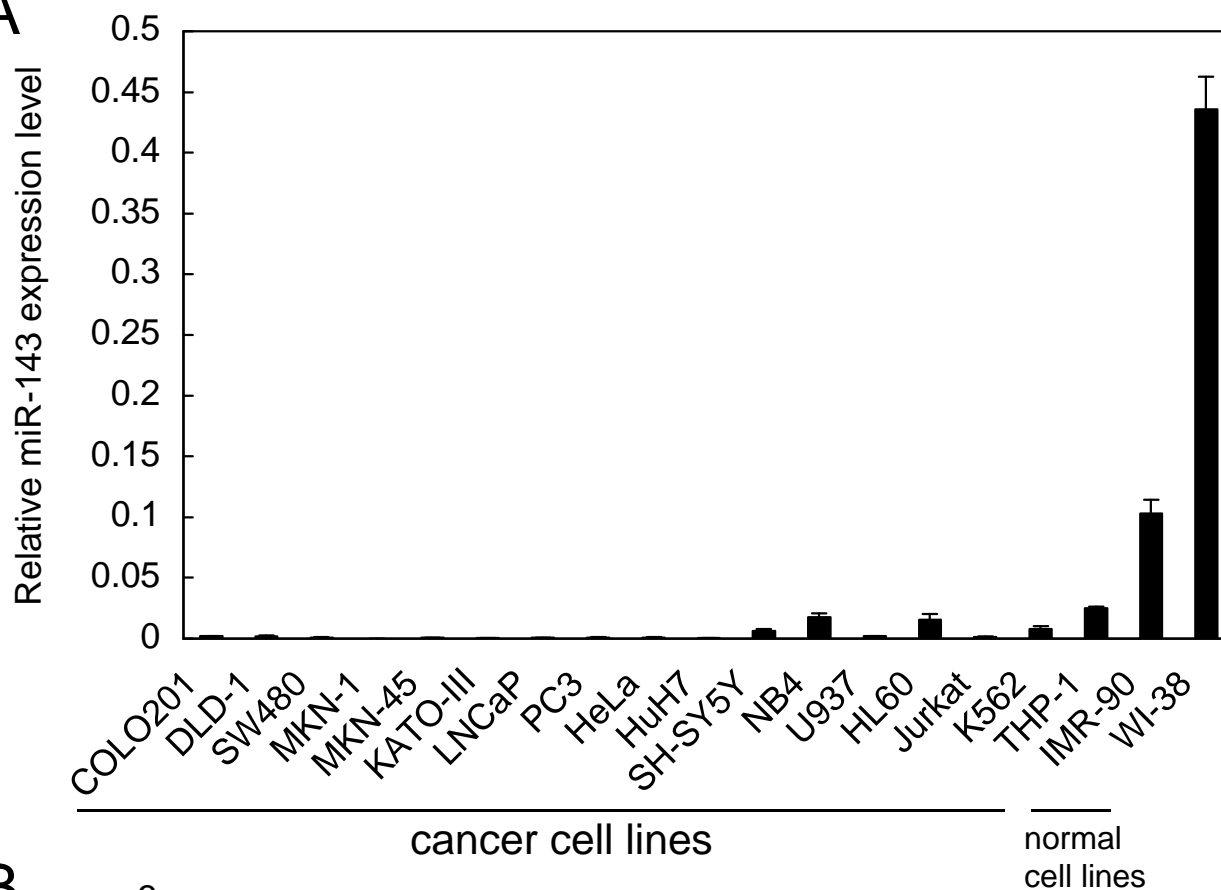

## B

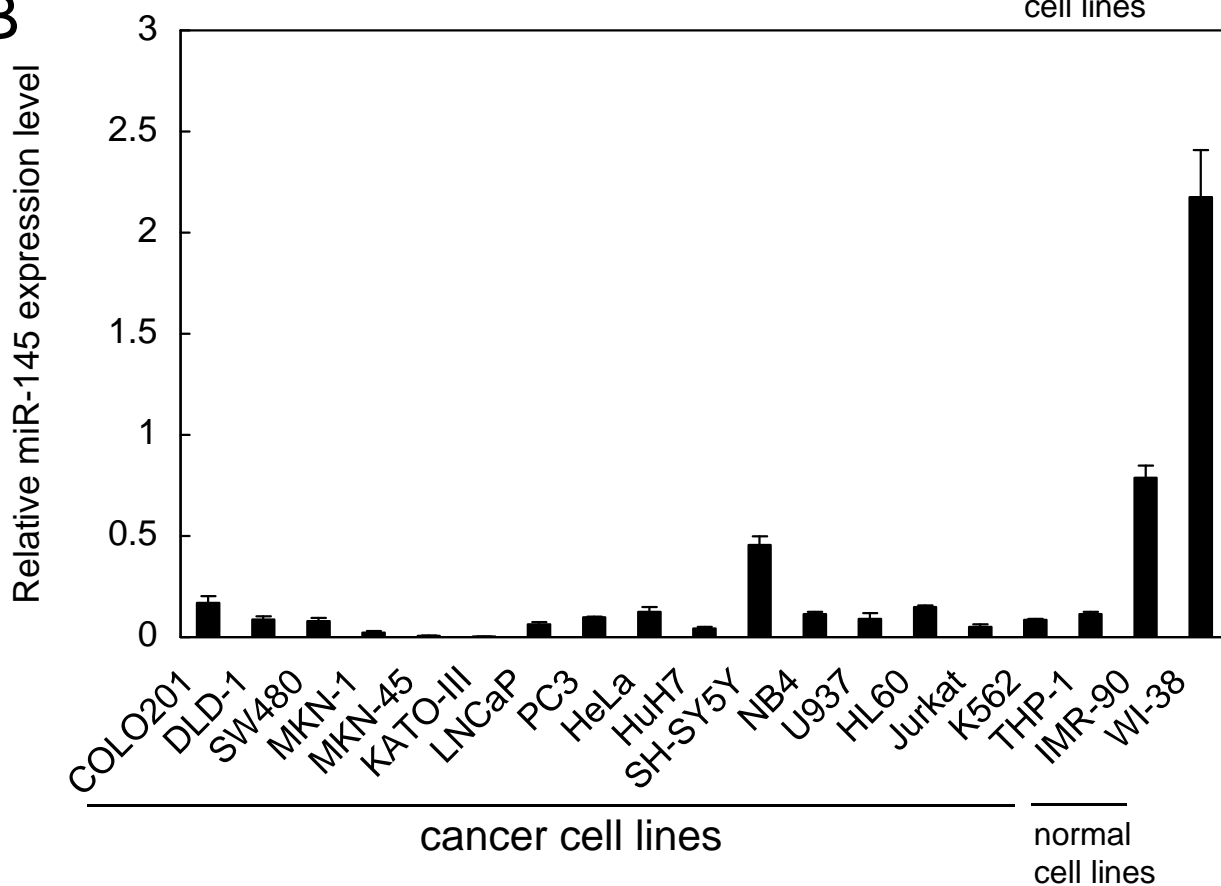

Figure S2

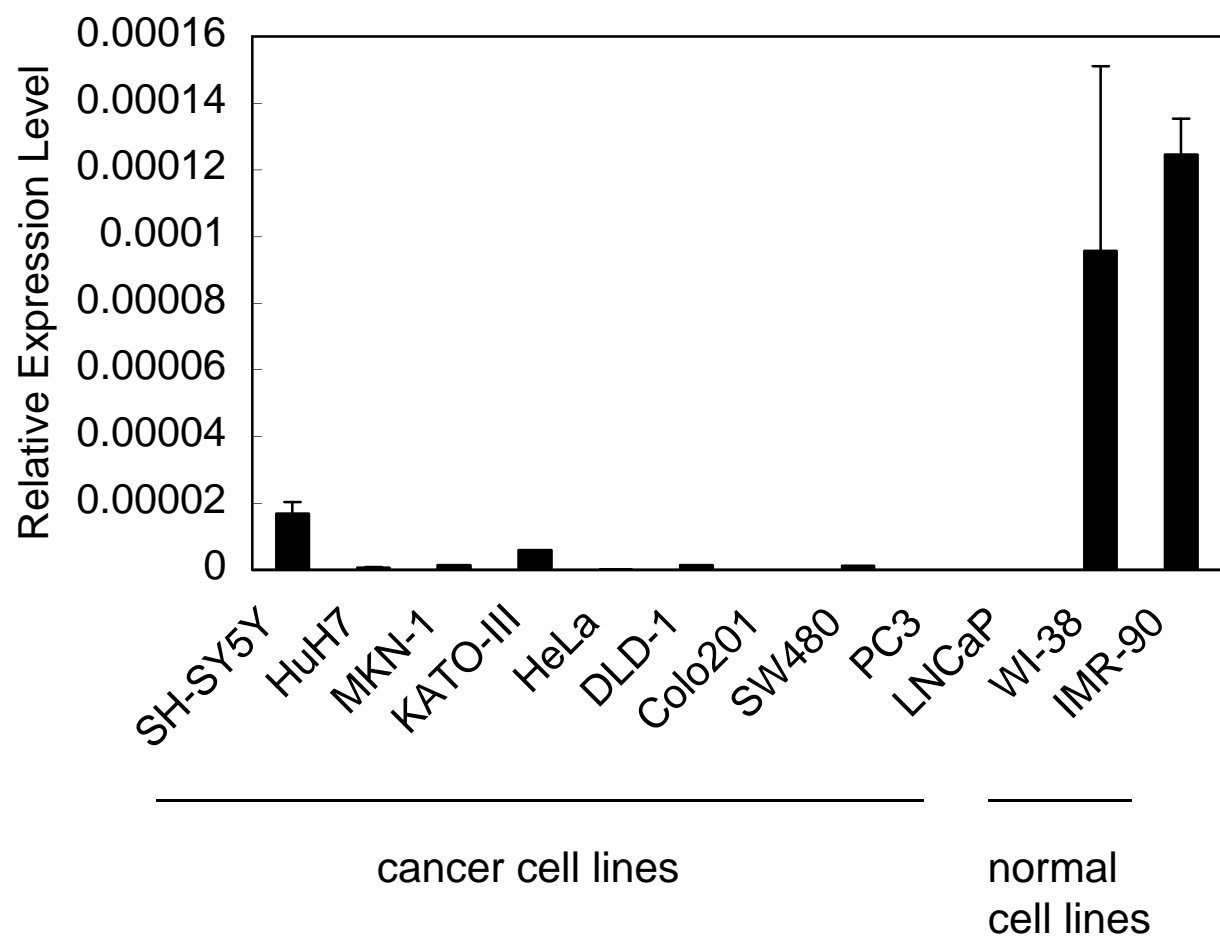

Figure S3

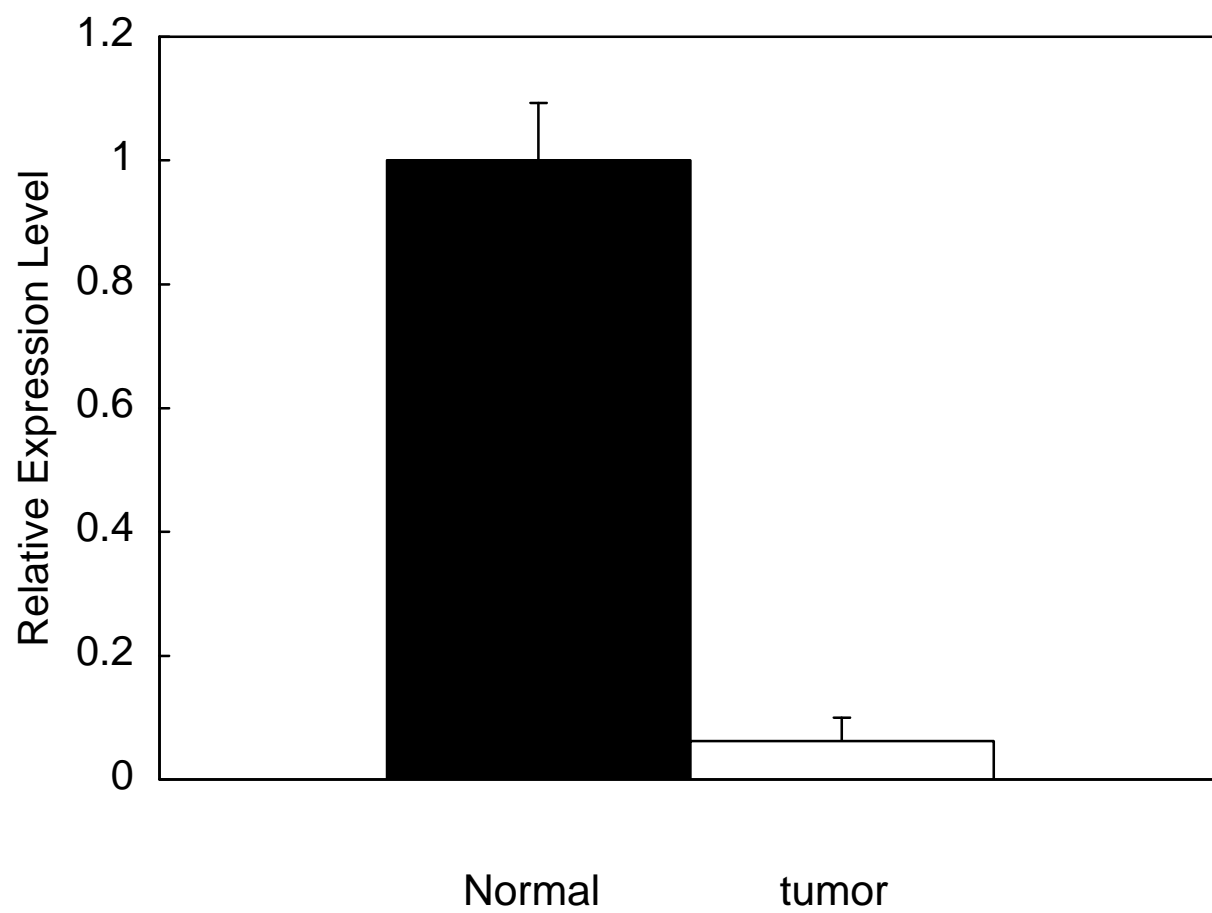

Figure S4

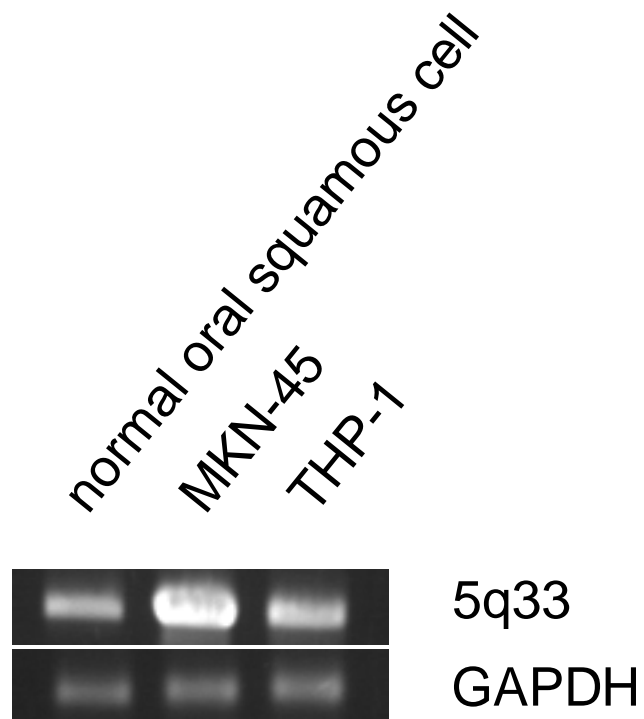

Supplement: Additional file 1 — Supplementary figures. Figure S1: Real-time RT-PCR analysis of mature miR-143 and -145 expression in human cell lines. Relative miR-143 (A) and -145 (B) expression levels are indicated on the left axis by using the comparative ΔCt method (value of 2-ΔCt(miR-RNU6B)). Figure S2: Real-time RT-PCR analysis of NCR143/145 expression in human cancer cell lines and normal cell lines. The relative expression level of NCR143/145 in human cancer cell lines was compared with that in human normal cell lines (WI-38 and IMR-90) by using the LOC10 primer set (see Fig. 2). Figure S3: Real-time RT-PCR analysis of NCR143/145 expression in human stomach. The relative expression level of NCR143/145 in human stomach tumor was compared with that in normal human stomach by using the LOC10 primer set (see Fig. 2). Figure S4: Confirmation of the presence of genomic loci of miR-143 and -145 at chromosome 5q33 by genomic PCR. We extracted genomic DNAs from 2 cell lines and normal human oral squamous cells by using DNAzol (Invitrogen, Carlsbad, USA) and used them for PCR. The 143-145 primer set was used for genomic loci of miRs-143 and -145 (see Fig. 2B). The genomic locus of GAPDH was used as an internal control. [file 1476-4598-9-136-S1.PDF]
